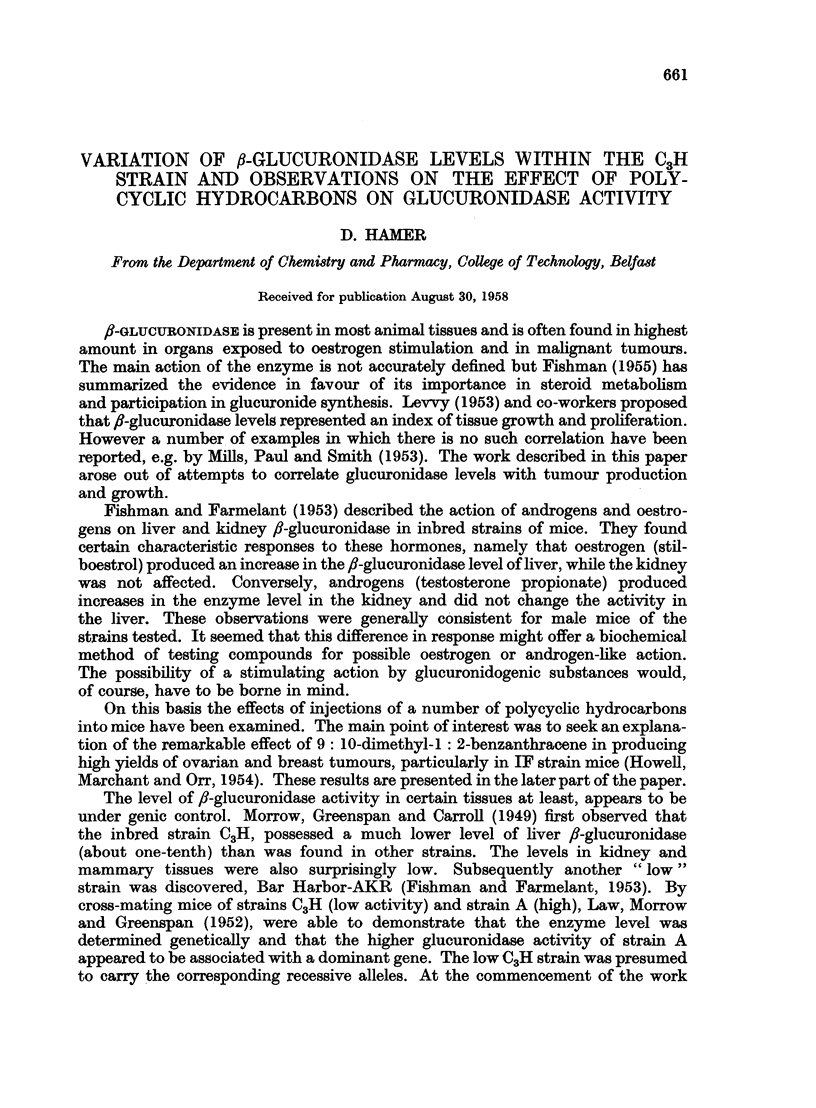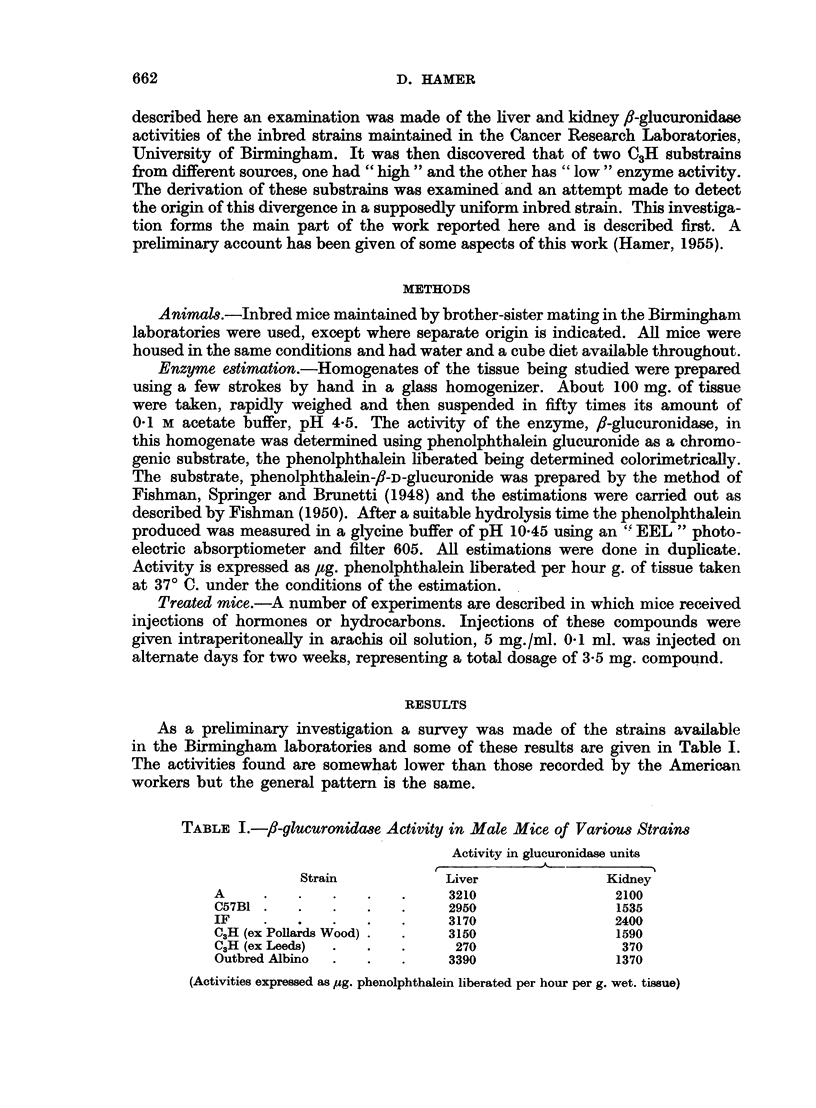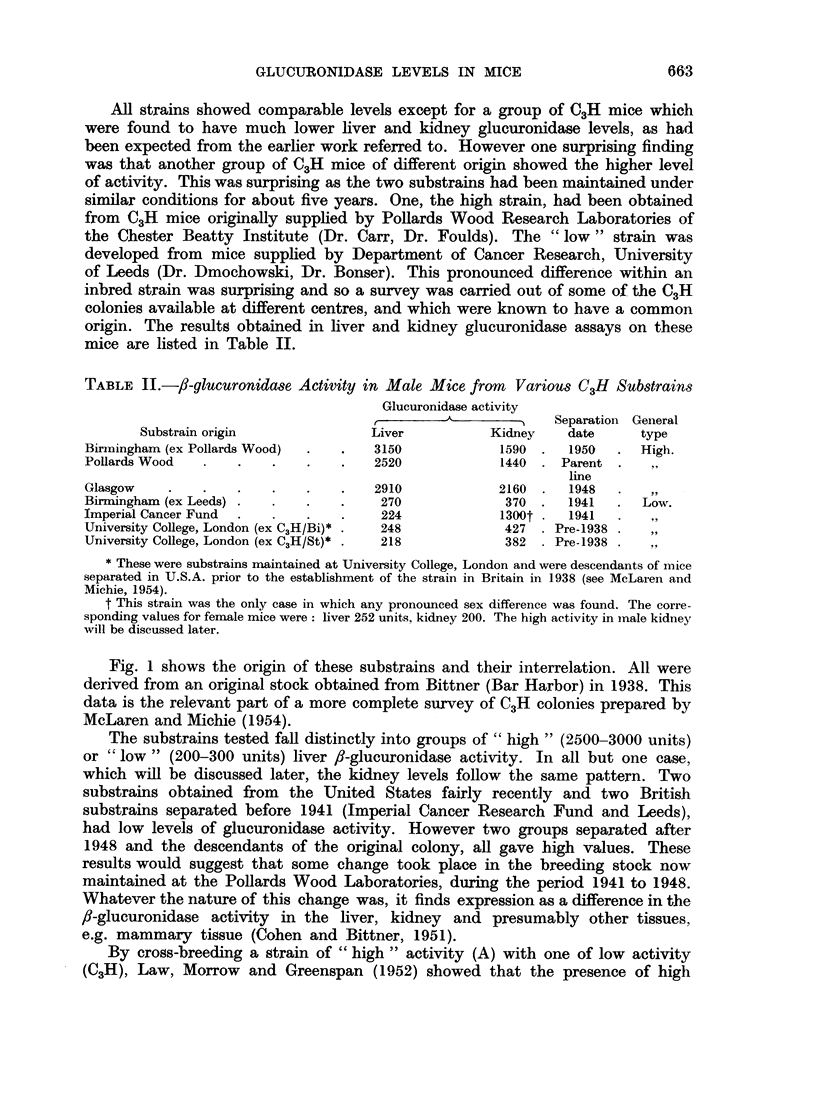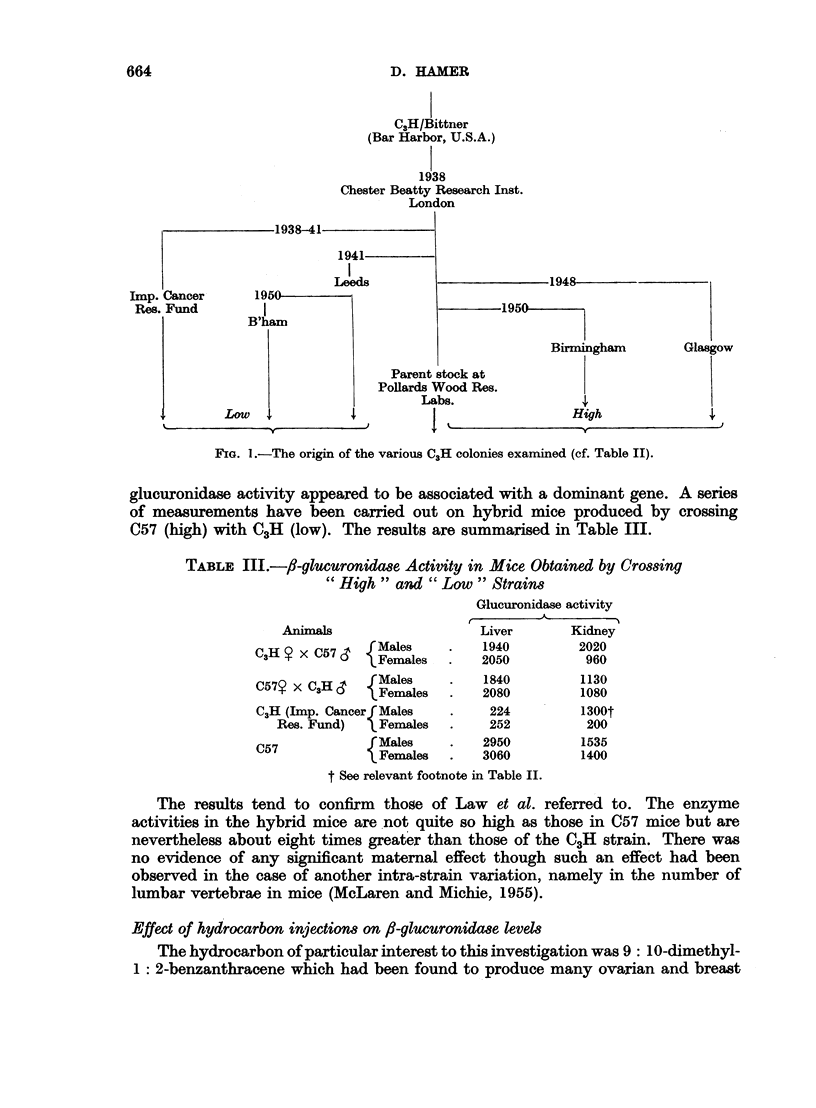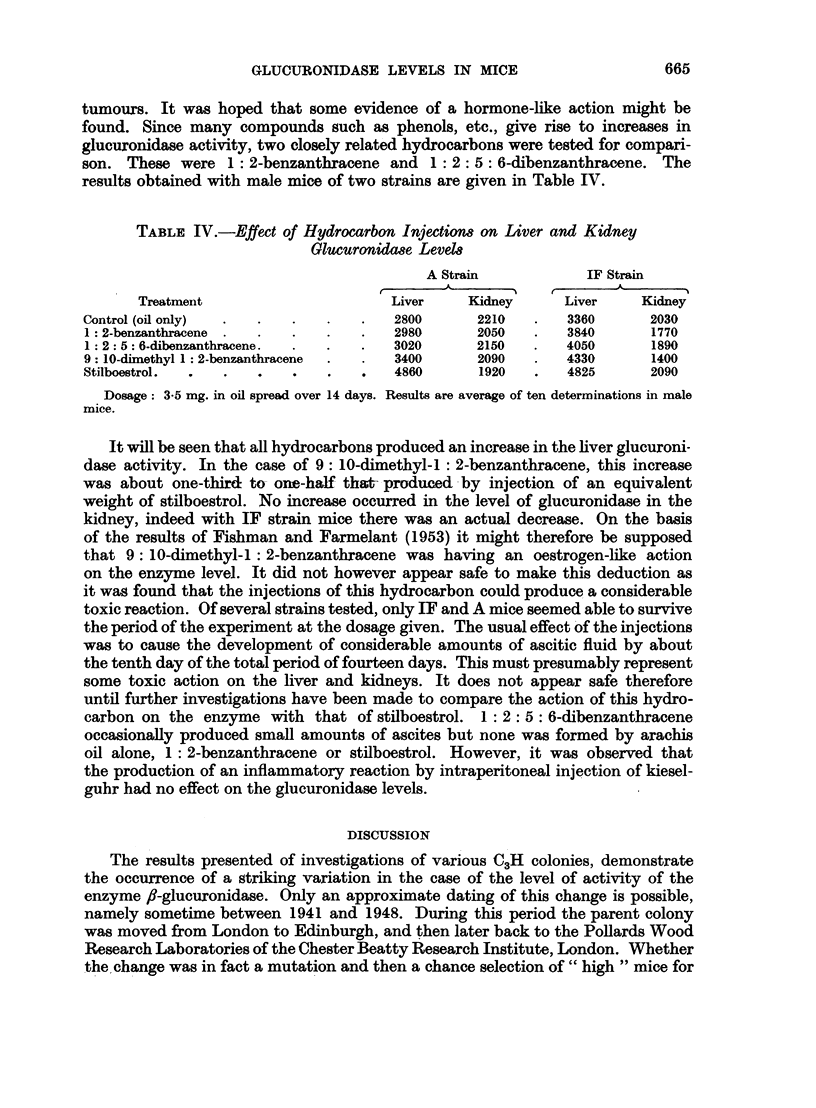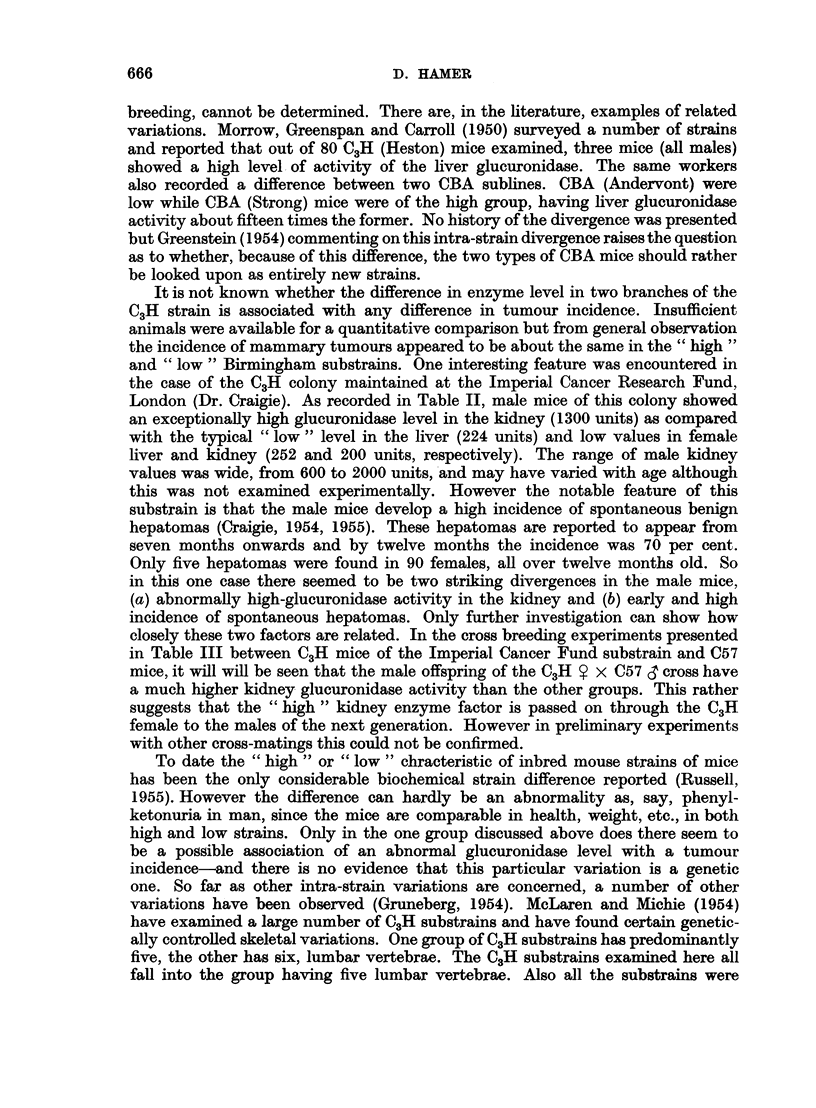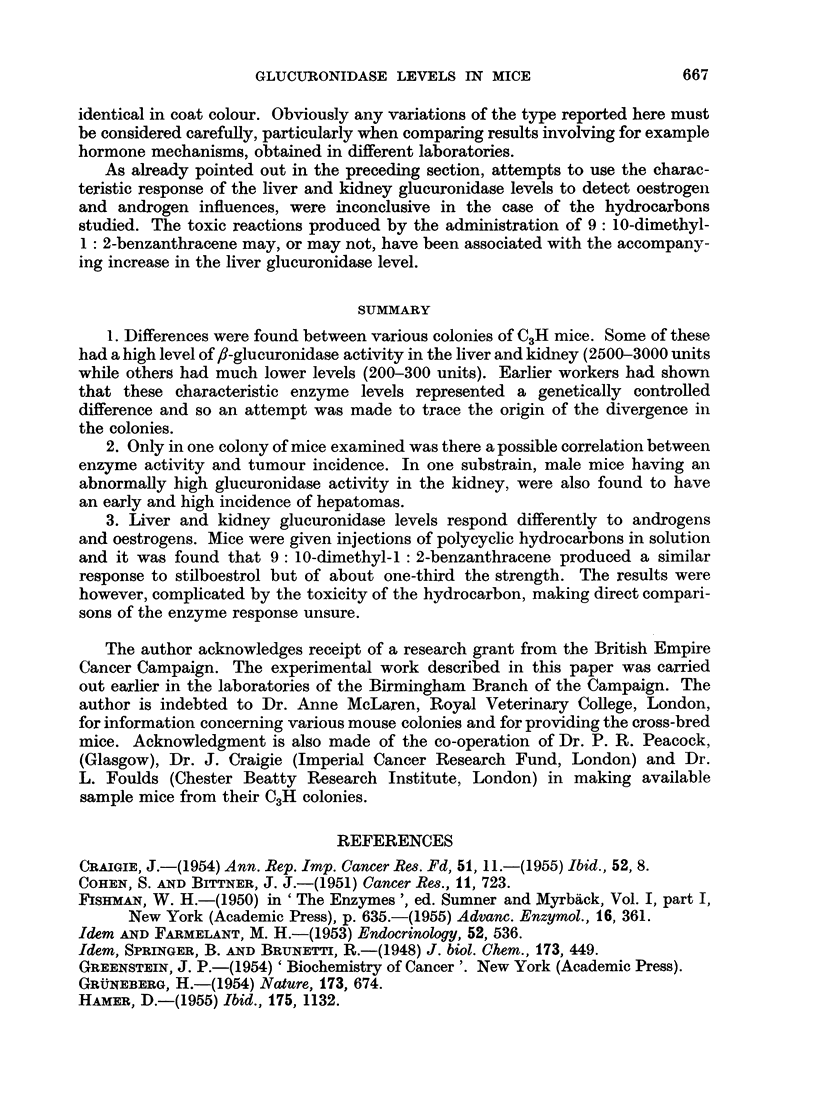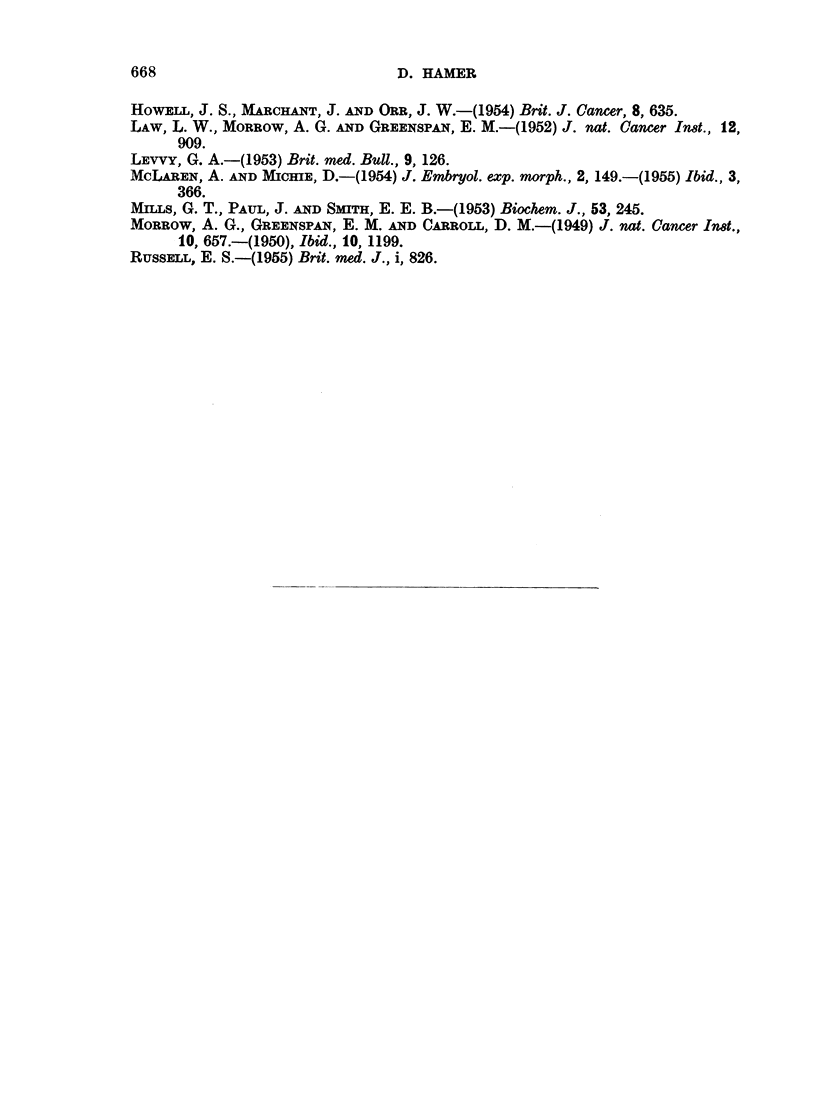# Variation of β-Glucuronidase Levels within the C_3_H Strain and Observations on the Effect of Polycyclic Hydrocarbons on Glucuronidase Activity

**DOI:** 10.1038/bjc.1958.74

**Published:** 1958-12

**Authors:** D. Hamer


### VARIATION OF $\beta$-GLUCURONIDASE LEVELS WITHIN THE $C_3H$ STRAIN AND OBSERVATIONS ON THE EFFECT OF POLYCYCLIC HYDROCARBONS ON GLUCURONIDASE ACTIVITY

D. HAMER

*From the Department of Chemistry and Pharmacy, College of Technology, Belfast*

Received for publication August 30, 1958

$\beta$-GLUCURONIDASE is present in most animal tissues and is often found in highest amount in organs exposed to oestrogen stimulation and in malignant tumours. The main action of the enzyme is not accurately defined but Fishman (1955) has summarized the evidence in favour of its importance in steroid metabolism and participation in glucuronide synthesis. Levvy (1953) and co-workers proposed that $\beta$-glucuronidase levels represented an index of tissue growth and proliferation. However a number of examples in which there is no such correlation have been reported, e.g. by Mills, Paul and Smith (1953). The work described in this paper arose out of attempts to correlate glucuronidase levels with tumour production and growth.

Fishman and Farmelant (1953) described the action of androgens and oestrogens on liver and kidney $\beta$-glucuronidase in inbred strains of mice. They found certain characteristic responses to these hormones, namely that oestrogen (stilboestrol) produced an increase in the $\beta$-glucuronidase level of liver, while the kidney was not affected. Conversely, androgens (testosterone propionate) produced increases in the enzyme level in the kidney and did not change the activity in the liver. These observations were generally consistent for male mice of the strains tested. It seemed that this difference in response might offer a biochemical method of testing compounds for possible oestrogen or androgen-like action. The possibility of a stimulating action by glucuronidogenic substances would, of course, have to be borne in mind.

On this basis the effects of injections of a number of polycyclic hydrocarbons into mice have been examined. The main point of interest was to seek an explanation of the remarkable effect of 9 : 10-dimethyl-1 : 2-benzanthracene in producing high yields of ovarian and breast tumours, particularly in IF strain mice (Howell, Marchant and Orr, 1954). These results are presented in the later part of the paper.

The level of $\beta$-glucuronidase activity in certain tissues at least, appears to be under genic control. Morrow, Greenspan and Carroll (1949) first observed that the inbred strain $C_3H$, possessed a much lower level of liver $\beta$-glucuronidase (about one-tenth) than was found in other strains. The levels in kidney and mammary tissues were also surprisingly low. Subsequently another "low" strain was discovered, Bar Harbor-AKR (Fishman and Farmelant, 1953). By cross-mating mice of strains $C_3H$ (low activity) and strain A (high), Law, Morrow and Greenspan (1952), were able to demonstrate that the enzyme level was determined genetically and that the higher glucuronidase activity of strain A appeared to be associated with a dominant gene. The low $C_3H$ strain was presumed to carry the corresponding recessive alleles. At the commencement of the work described here an examination was made of the liver and kidney β-glucuronidase activities of the inbred strains maintained in the Cancer Research Laboratories, University of Birmingham. It was then discovered that of two $C_3H$ substrains from different sources, one had " high " and the other has " low " enzyme activity. The derivation of these substrains was examined and an attempt made to detect the origin of this divergence in a supposedly uniform inbred strain. This investigation forms the main part of the work reported here and is described first. A preliminary account has been given of some aspects of this work (Hamer, 1955).

### METHODS

*Animals.*—Inbred mice maintained by brother-sister mating in the Birmingham laboratories were used, except where separate origin is indicated. All mice were housed in the same conditions and had water and a cube diet available throughout.

*Enzyme estimation.*—Homogenates of the tissue being studied were prepared using a few strokes by hand in a glass homogenizer. About 100 mg. of tissue were taken, rapidly weighed and then suspended in fifty times its amount of 0·1 M acetate buffer, pH 4·5. The activity of the enzyme, β-glucuronidase, in this homogenate was determined using phenolphthalein glucuronide as a chromogenic substrate, the phenolphthalein liberated being determined colorimetrically. The substrate, phenolphthalein-β-D-glucuronide was prepared by the method of Fishman, Springer and Brunetti (1948) and the estimations were carried out as described by Fishman (1950). After a suitable hydrolysis time the phenolphthalein produced was measured in a glycine buffer of pH 10·45 using an " EEL " photoelectric absorptiometer and filter 605. All estimations were done in duplicate. Activity is expressed as μg. phenolphthalein liberated per hour g. of tissue taken at 37° C. under the conditions of the estimation.

*Treated mice.*—A number of experiments are described in which mice received injections of hormones or hydrocarbons. Injections of these compounds were given intraperitoneally in arachis oil solution, 5 mg./ml. 0·1 ml. was injected on alternate days for two weeks, representing a total dosage of 3·5 mg. compound.

### RESULTS

As a preliminary investigation a survey was made of the strains available in the Birmingham laboratories and some of these results are given in Table I. The activities found are somewhat lower than those recorded by the American workers but the general pattern is the same.

TABLE I.—*β-glucuronidase Activity in Male Mice of Various Strains*

| Strain | Activity in glucuronidase units: Liver | Kidney |
|---|---|---|
| A | 3210 | 2100 |
| C57Bl | 2950 | 1535 |
| IF | 3170 | 2400 |
| $C_3H$ (ex Pollards Wood) | 3150 | 1590 |
| $C_3H$ (ex Leeds) | 270 | 370 |
| Outbred Albino | 3390 | 1370 |

(Activities expressed as μg. phenolphthalein liberated per hour per g. wet. tissue)

All strains showed comparable levels except for a group of $C_3H$ mice which were found to have much lower liver and kidney glucuronidase levels, as had been expected from the earlier work referred to. However one surprising finding was that another group of $C_3H$ mice of different origin showed the higher level of activity. This was surprising as the two substrains had been maintained under similar conditions for about five years. One, the high strain, had been obtained from $C_3H$ mice originally supplied by Pollards Wood Research Laboratories of the Chester Beatty Institute (Dr. Carr, Dr. Foulds). The " low " strain was developed from mice supplied by Department of Cancer Research, University of Leeds (Dr. Dmochowski, Dr. Bonser). This pronounced difference within an inbred strain was surprising and so a survey was carried out of some of the $C_3H$ colonies available at different centres, and which were known to have a common origin. The results obtained in liver and kidney glucuronidase assays on these mice are listed in Table II.

TABLE II.—*β-glucuronidase Activity in Male Mice from Various $C_3H$ Substrains*

| Substrain origin | Glucuronidase activity: Liver | Glucuronidase activity: Kidney | Separation date | General type |
|---|---|---|---|---|
| Birmingham (ex Pollards Wood) | 3150 | 1590 | 1950 | High. |
| Pollards Wood | 2520 | 1440 | Parent line | ,, |
| Glasgow | 2910 | 2160 | 1948 | ,, |
| Birmingham (ex Leeds) | 270 | 370 | 1941 | Low. |
| Imperial Cancer Fund | 224 | 1300† | 1941 | ,, |
| University College, London (ex $C_3H$/Bi)* | 248 | 427 | Pre-1938 | ,, |
| University College, London (ex $C_3H$/St)* | 218 | 382 | Pre-1938 | ,, |

* These were substrains maintained at University College, London and were descendants of mice separated in U.S.A. prior to the establishment of the strain in Britain in 1938 (see McLaren and Michie, 1954).

† This strain was the only case in which any pronounced sex difference was found. The corresponding values for female mice were : liver 252 units, kidney 200. The high activity in male kidney will be discussed later.

Fig. 1 shows the origin of these substrains and their interrelation. All were derived from an original stock obtained from Bittner (Bar Harbor) in 1938. This data is the relevant part of a more complete survey of $C_3H$ colonies prepared by McLaren and Michie (1954).

The substrains tested fall distinctly into groups of " high " (2500–3000 units) or " low " (200–300 units) liver β-glucuronidase activity. In all but one case, which will be discussed later, the kidney levels follow the same pattern. Two substrains obtained from the United States fairly recently and two British substrains separated before 1941 (Imperial Cancer Research Fund and Leeds), had low levels of glucuronidase activity. However two groups separated after 1948 and the descendants of the original colony, all gave high values. These results would suggest that some change took place in the breeding stock now maintained at the Pollards Wood Laboratories, during the period 1941 to 1948. Whatever the nature of this change was, it finds expression as a difference in the β-glucuronidase activity in the liver, kidney and presumably other tissues, e.g. mammary tissue (Cohen and Bittner, 1951).

By cross-breeding a strain of " high " activity (A) with one of low activity ($C_3H$), Law, Morrow and Greenspan (1952) showed that the presence of high

FIG. 1.—The origin of the various $C_3H$ colonies examined (cf. Table II).

glucuronidase activity appeared to be associated with a dominant gene. A series of measurements have been carried out on hybrid mice produced by crossing C57 (high) with $C_3H$ (low). The results are summarised in Table III.

TABLE III.—*β-glucuronidase Activity in Mice Obtained by Crossing "High" and "Low" Strains*

| Animals | | Glucuronidase activity: Liver | Glucuronidase activity: Kidney |
|---|---|---|---|
| $C_3H$ ♀ × C57 ♂ | Males | 1940 | 2020 |
| | Females | 2050 | 960 |
| C57♀ × $C_3H$ ♂ | Males | 1840 | 1130 |
| | Females | 2080 | 1080 |
| $C_3H$ (Imp. Cancer Res. Fund) | Males | 224 | 1300† |
| | Females | 252 | 200 |
| C57 | Males | 2950 | 1535 |
| | Females | 3060 | 1400 |

† See relevant footnote in Table II.

The results tend to confirm those of Law *et al.* referred to. The enzyme activities in the hybrid mice are not quite so high as those in C57 mice but are nevertheless about eight times greater than those of the $C_3H$ strain. There was no evidence of any significant maternal effect though such an effect had been observed in the case of another intra-strain variation, namely in the number of lumbar vertebrae in mice (McLaren and Michie, 1955).

*Effect of hydrocarbon injections on β-glucuronidase levels*

The hydrocarbon of particular interest to this investigation was 9 : 10-dimethyl-1 : 2-benzanthracene which had been found to produce many ovarian and breast tumours. It was hoped that some evidence of a hormone-like action might be found. Since many compounds such as phenols, etc., give rise to increases in glucuronidase activity, two closely related hydrocarbons were tested for comparison. These were 1 : 2-benzanthracene and 1 : 2 : 5 : 6-dibenzanthracene. The results obtained with male mice of two strains are given in Table IV.

TABLE IV.—*Effect of Hydrocarbon Injections on Liver and Kidney Glucuronidase Levels*

| | A Strain | | IF Strain | |
|---|---|---|---|---|
| Treatment | Liver | Kidney | Liver | Kidney |
| Control (oil only) | 2800 | 2210 | 3360 | 2030 |
| 1 : 2-benzanthracene | 2980 | 2050 | 3840 | 1770 |
| 1 : 2 : 5 : 6-dibenzanthracene | 3020 | 2150 | 4050 | 1890 |
| 9 : 10-dimethyl 1 : 2-benzanthracene | 3400 | 2090 | 4330 | 1400 |
| Stilboestrol | 4860 | 1920 | 4825 | 2090 |

Dosage : 3·5 mg. in oil spread over 14 days. Results are average of ten determinations in male mice.

It will be seen that all hydrocarbons produced an increase in the liver glucuronidase activity. In the case of 9 : 10-dimethyl-1 : 2-benzanthracene, this increase was about one-third to one-half that produced by injection of an equivalent weight of stilboestrol. No increase occurred in the level of glucuronidase in the kidney, indeed with IF strain mice there was an actual decrease. On the basis of the results of Fishman and Farmelant (1953) it might therefore be supposed that 9 : 10-dimethyl-1 : 2-benzanthracene was having an oestrogen-like action on the enzyme level. It did not however appear safe to make this deduction as it was found that the injections of this hydrocarbon could produce a considerable toxic reaction. Of several strains tested, only IF and A mice seemed able to survive the period of the experiment at the dosage given. The usual effect of the injections was to cause the development of considerable amounts of ascitic fluid by about the tenth day of the total period of fourteen days. This must presumably represent some toxic action on the liver and kidneys. It does not appear safe therefore until further investigations have been made to compare the action of this hydrocarbon on the enzyme with that of stilboestrol. 1 : 2 : 5 : 6-dibenzanthracene occasionally produced small amounts of ascites but none was formed by arachis oil alone, 1 : 2-benzanthracene or stilboestrol. However, it was observed that the production of an inflammatory reaction by intraperitoneal injection of kieselguhr had no effect on the glucuronidase levels.

### DISCUSSION

The results presented of investigations of various $C_3H$ colonies, demonstrate the occurrence of a striking variation in the case of the level of activity of the enzyme $\beta$-glucuronidase. Only an approximate dating of this change is possible, namely sometime between 1941 and 1948. During this period the parent colony was moved from London to Edinburgh, and then later back to the Pollards Wood Research Laboratories of the Chester Beatty Research Institute, London. Whether the change was in fact a mutation and then a chance selection of " high " mice for breeding, cannot be determined. There are, in the literature, examples of related variations. Morrow, Greenspan and Carroll (1950) surveyed a number of strains and reported that out of 80 $C_3H$ (Heston) mice examined, three mice (all males) showed a high level of activity of the liver glucuronidase. The same workers also recorded a difference between two CBA sublines. CBA (Andervont) were low while CBA (Strong) mice were of the high group, having liver glucuronidase activity about fifteen times the former. No history of the divergence was presented but Greenstein (1954) commenting on this intra-strain divergence raises the question as to whether, because of this difference, the two types of CBA mice should rather be looked upon as entirely new strains.

It is not known whether the difference in enzyme level in two branches of the $C_3H$ strain is associated with any difference in tumour incidence. Insufficient animals were available for a quantitative comparison but from general observation the incidence of mammary tumours appeared to be about the same in the " high " and " low " Birmingham substrains. One interesting feature was encountered in the case of the $C_3H$ colony maintained at the Imperial Cancer Research Fund, London (Dr. Craigie). As recorded in Table II, male mice of this colony showed an exceptionally high glucuronidase level in the kidney (1300 units) as compared with the typical " low " level in the liver (224 units) and low values in female liver and kidney (252 and 200 units, respectively). The range of male kidney values was wide, from 600 to 2000 units, and may have varied with age although this was not examined experimentally. However the notable feature of this substrain is that the male mice develop a high incidence of spontaneous benign hepatomas (Craigie, 1954, 1955). These hepatomas are reported to appear from seven months onwards and by twelve months the incidence was 70 per cent. Only five hepatomas were found in 90 females, all over twelve months old. So in this one case there seemed to be two striking divergences in the male mice, (*a*) abnormally high-glucuronidase activity in the kidney and (*b*) early and high incidence of spontaneous hepatomas. Only further investigation can show how closely these two factors are related. In the cross breeding experiments presented in Table III between $C_3H$ mice of the Imperial Cancer Fund substrain and C57 mice, it will will be seen that the male offspring of the $C_3H$ ♀ × C57 ♂ cross have a much higher kidney glucuronidase activity than the other groups. This rather suggests that the " high " kidney enzyme factor is passed on through the $C_3H$ female to the males of the next generation. However in preliminary experiments with other cross-matings this could not be confirmed.

To date the " high " or " low " chracteristic of inbred mouse strains of mice has been the only considerable biochemical strain difference reported (Russell, 1955). However the difference can hardly be an abnormality as, say, phenylketonuria in man, since the mice are comparable in health, weight, etc., in both high and low strains. Only in the one group discussed above does there seem to be a possible association of an abnormal glucuronidase level with a tumour incidence—and there is no evidence that this particular variation is a genetic one. So far as other intra-strain variations are concerned, a number of other variations have been observed (Gruneberg, 1954). McLaren and Michie (1954) have examined a large number of $C_3H$ substrains and have found certain genetically controlled skeletal variations. One group of $C_3H$ substrains has predominantly five, the other has six, lumbar vertebrae. The $C_3H$ substrains examined here all fall into the group having five lumbar vertebrae. Also all the substrains were identical in coat colour. Obviously any variations of the type reported here must be considered carefully, particularly when comparing results involving for example hormone mechanisms, obtained in different laboratories.

As already pointed out in the preceding section, attempts to use the characteristic response of the liver and kidney glucuronidase levels to detect oestrogen and androgen influences, were inconclusive in the case of the hydrocarbons studied. The toxic reactions produced by the administration of 9 : 10-dimethyl-1 : 2-benzanthracene may, or may not, have been associated with the accompanying increase in the liver glucuronidase level.

### SUMMARY

1. Differences were found between various colonies of $C_3H$ mice. Some of these had a high level of $\beta$-glucuronidase activity in the liver and kidney (2500–3000 units while others had much lower levels (200–300 units). Earlier workers had shown that these characteristic enzyme levels represented a genetically controlled difference and so an attempt was made to trace the origin of the divergence in the colonies.

2. Only in one colony of mice examined was there a possible correlation between enzyme activity and tumour incidence. In one substrain, male mice having an abnormally high glucuronidase activity in the kidney, were also found to have an early and high incidence of hepatomas.

3. Liver and kidney glucuronidase levels respond differently to androgens and oestrogens. Mice were given injections of polycyclic hydrocarbons in solution and it was found that 9 : 10-dimethyl-1 : 2-benzanthracene produced a similar response to stilboestrol but of about one-third the strength. The results were however, complicated by the toxicity of the hydrocarbon, making direct comparisons of the enzyme response unsure.

The author acknowledges receipt of a research grant from the British Empire Cancer Campaign. The experimental work described in this paper was carried out earlier in the laboratories of the Birmingham Branch of the Campaign. The author is indebted to Dr. Anne McLaren, Royal Veterinary College, London, for information concerning various mouse colonies and for providing the cross-bred mice. Acknowledgment is also made of the co-operation of Dr. P. R. Peacock, (Glasgow), Dr. J. Craigie (Imperial Cancer Research Fund, London) and Dr. L. Foulds (Chester Beatty Research Institute, London) in making available sample mice from their $C_3H$ colonies.

### REFERENCES

Craigie, J.—(1954) *Ann. Rep. Imp. Cancer Res. Fd*, **51**, 11.—(1955) *Ibid.*, **52**, 8.
Cohen, S. and Bittner, J. J.—(1951) *Cancer Res.*, **11**, 723.
Fishman, W. H.—(1950) in ‘ The Enzymes ’, ed. Sumner and Myrbäck, Vol. I, part I, New York (Academic Press), p. 635.—(1955) *Advanc. Enzymol.*, **16**, 361.
*Idem* and Farmelant, M. H.—(1953) *Endocrinology*, **52**, 536.
*Idem*, Springer, B. and Brunetti, R.—(1948) *J. biol. Chem.*, **173**, 449.
Greenstein, J. P.—(1954) ‘ Biochemistry of Cancer ’. New York (Academic Press).
Grüneberg, H.—(1954) *Nature*, **173**, 674.
Hamer, D.—(1955) *Ibid.*, **175**, 1132.

Howell, J. S., Marchant, J. and Orr, J. W.—(1954) *Brit. J. Cancer*, **8**, 635.
Law, L. W., Morrow, A. G. and Greenspan, E. M.—(1952) *J. nat. Cancer Inst.*, **12**, 909.
Levvy, G. A.—(1953) *Brit. med. Bull.*, **9**, 126.
McLaren, A. and Michie, D.—(1954) *J. Embryol. exp. morph.*, **2**, 149.—(1955) *Ibid.*, **3**, 366.
Mills, G. T., Paul, J. and Smith, E. E. B.—(1953) *Biochem. J.*, **53**, 245.
Morrow, A. G., Greenspan, E. M. and Carroll, D. M.—(1949) *J. nat. Cancer Inst.*, **10**, 657.—(1950), *Ibid.*, **10**, 1199.
Russell, E. S.—(1955) *Brit. med. J.*, i, 826.